# Investigating the efficacy of a varnish containing gallic acid on remineralization of enamel lesions: an in vitro study

**DOI:** 10.1186/s12903-024-03921-7

**Published:** 2024-02-03

**Authors:** Iman Parisay, Marzie Boskabady, Hossein Bagheri, Saber Babazadeh, Melika Hoseinzadeh, Fatemeh Esmaeilzadeh

**Affiliations:** 1 Department of Pediatric Dentistry, Faculty of Dentistry, Mashhad University of Medical Sciences, Mashhad, Iran; 2 Dental Materials Research Center, Faculty of Dentistry, Mashhad University of Medical Sciences, Mashhad, Iran; 3 Department of Community Oral Health, Faculty of Dentistry, Mashhad University of Medical Sciences, Mashhad, Iran; 4 Dental caries Prevention Research Center, Faculty of Dentistry, Qazvin University Medical Sciences, Qazvin, Iran; 5 Dental Research Center, Mashhad Dental School, Mashhad University of Medical Sciences, Mashhad, Iran

**Keywords:** Fluoride, Gallic acid, Microhardness test, Tooth remineralization

## Abstract

This study evaluated the efficacy of a formulated remineralizing gallic acid (GA) varnish in treating artificial enamel caries lesions. Fifty-five intact bovine incisors were collected. Enamel blocks (5 × 9 mm) were prepared. A third of each block’s surface remained intact. Primary carious lesions were induced on the middle and bottom thirds of the blocks by immersing the samples in a demineralization solution for 6 h. The bottom third of the blocks were further remineralized by randomly applying 0.5%, 2%, or 8% GA varnishes and 2.26% fluoride varnish (V varnish, Vericom, Seoul, Korea), or the varnish base without active ingredients (*n* = 11 each). The specimens were immersed in a remineralizing solution for 4 h and then subjected to a 2-hour immersion in the demineralizing solution. After six days of pH cycling, the surface microhardness was measured at depths of 30, 75, and 120 μm. The percentage of surface microhardness recovery (SMHR%) was compared among the groups using the Shapiro-Wilk, ANOVA, and Tukey HSD post-hoc tests (α = 0.05). The SMHR% of all experimental groups was higher than the control group at 30 μm (*p* < 0.05). The 0.5% GA varnish showed the highest SMHR% at all depths; however, the difference with the other experimental groups was significant at a depth of 30 μm (*p* < 0.05). The SMHR% of the fluoride and the 2% and 8% GA varnishes was comparable at all depths. All treatments potentially remineralize enamel lesions, with 0.5% GA varnish having the greatest effect, particularly on the top surface layer. As such, this newly developed varnish may emerge as a promising alternative to fluoride varnish.

## Introduction

Dental caries is a prevalent concern in children’s oral health, yet its prevention remains straightforward. A widely employed strategy to prevent dental caries involves applying fluoride varnish 2–4 times annually to both primary and permanent teeth [1]. However, the fluoride varnish application encounters limitations due to its possible side effects. Some individuals may experience heightened sensitivity and a burning sensation in the mouth. Additionally, the possibility of fluorosis exists in regions where drinking water contains elevated fluoride concentrations [2]. Furthermore, excessive fluoride consumption can result in symptoms like nausea, vomiting, diarrhea, escalated salivation, abdominal discomfort, muscle frailty, and spasms [3]. Fluoride varnish is not recommended for patients with ulcerative gingivitis, gingivitis stomatitis, or asthma [4, 5]. Additionally, concerns regarding the safety and efficacy of topical fluoride among caregivers have led to reluctance or refusal of its application on children [6].

Hence, researchers have explored the potential of bioactive herbal components in preventing demineralization and promoting remineralization. Gallic acid (GA), a natural polyphenol in diverse plants, has emerged as a promising candidate. Its accessibility and the relatively uncomplicated and cost-effective extraction process underscore its potential. GA boasts a spectrum of biological attributes encompassing anti-inflammatory, antioxidant, antiviral, and antibacterial effects against cariogenic bacteria [7]. Extensive research highlights the remineralizing effect of medicinal plants that house GA, such as *Galla chinensis* – a traditional Chinese medicine known for preventing demineralization, facilitating remineralization, and having synergism with fluoride and nanohydroxyapatite [8–11]. GA in grape seeds further supports remineralization while mitigating dentinal hypersensitivity [12, 13]. *Quercus infectoria* galls, rich in tannic acid and GA, have been shown to prevent demineralization and enhance remineralization [14]. Extracts from Pistacia lentiscus prove more effective than 1000 ppm sodium fluoride in promoting dentinal tubule closure and hydroxyapatite crystal remineralization [15]. Zou et al. [16] showed that *Galla chinensis* extract decreases demineralization under dynamic pH cycling conditions. It is hypnotized that GA’s strong bonding to the enamel matrix proteins and Ca is believed to drive hydroxyapatite crystal nucleation on carious lesion surfaces and enhance resistance to demineralization [9, 10, 14, 17]. Moreover, GA combines with nanohydroxyapatite and modifies hydroxyapatite crystal morphology and structure, hence remineralizing bleached enamel [13, 18].

Existing studies have predominantly delved into GA within plant extracts, overlooking GA’s isolated form. While several studies have explored the effects of GA in aqueous solutions with favorable outcomes, the formulation of a varnish exposing teeth to a high GA concentration has remained uncharted territory. Many people desire to use herbal oral health products compared to synthetic products [19]. Therefore, there is a need to provide an alternative to fluoride varnish. Hence, this study aimed to synthesize GA varnishes with different concentrations and compare their remineralization effects to fluoride varnish by measuring the surface microhardness recovery values.

## Methods and materials

The protocol of this in vitro study was approved by the Ethics Committee of Mashhad University of Medical Sciences (code: IR.MUMS.DENTISTRY.REC.1401.054).

### Formulation of gallic acid varnish

A varnish base was prepared by mixing rosin gum (Shokouh Parvaz, Iran), polyvinyl acetate (PVA) (Titrachem, Iran), and silica (Arkachem, Iran) with a magnetic stirrer (Behsan, Iran) at room temperature for 24 h. Subsequently, Gallic Acid (GA) was added in 0.5%, 2%, and 8% w/w concentrations. The mixture was stirred in a closed container at room temperature for an additional hour [20]. The final formulation of the GA varnish consisted of 2 g of ethanol, 0.25 g of rosin gum, 0.25 g of PVA, 0.1 g of silica, and GA in concentrations of 0.5%, 2%, and 8% w/w.

### Sample collection and preparation

The sample size was determined using G*Power based on findings from Chu et al. [21], with an effect size of 0.5, a power of 0.8, and a significance level of 0.05. The resulting sample size was 11 per group. Fifty-five intact bovine central teeth, extracted within the last three months, were collected, rinsed under tap water, and stored at 4 °C in 0.05% thymol water until the investigation started [8].

The roots of the samples were removed, and 5 × 9 mm blocks were obtained from the mid-buccal area of the crowns using a CNC cutting machine (LamPlan, Iran). The enamel blocks were mounted in self-curing acrylic (Acroopars, Iran), exposing the buccal surfaces. Samples were then polished with silicon carbide sandpaper of grit sizes 400 to 1000 (Matador, Germany) to ensure a smooth surface.

### Primary carious lesion formation

A demineralizing solution was prepared with 2.2 mmol/L CaCl2, 2.2 mmol/L Na2HPO4, 50 mmol/L acetic acid, and 0.2 mmol/L sodium benzoate. The solution’s pH was adjusted to 4.5 using 1 mol/L NaOH, and the volume was brought to 1000 cc with Deuterium-depleted water (DDW) distilled water [22]. Since most studies have focused on the effectiveness of remieneralizing varnishes on primary human teeth, three experimental pilots were conducted to determine the optimal demineralization composition and duration. The samples were selected based on the same inclusion criteria as the study groups’ samples. While the microhardness measurement of the main groups was performed in different depths after cross-sectioning, the measurements of the pilot samples were conducted on the enamel surface:

- Pilot 1: Five samples were exposed to the demineralizing solution without NaF for 4 h, resulting in a significant decrease in surface microhardness by about 90–100 kg/mm2.
- Pilot 2: Five samples were subjected to the demineralizing solution without NaF for 10–12 h. Despite a microhardness reduction of at least 100 μm, the enamel surface was destroyed due to prolonged demineralization.
- Pilot 3: Based on the findings of Nozari et al. [23], 0.1 ppm NaF was added to protect the enamel surface layer. Five samples were immersed in the solution for 6 h. Microhardness tests showed a reduction of 130–205 units, and the enamel surface remained intact. The enamel surface intactness was determined under the Vickers microhardness tester device’s microscope (x200) by the sharpness and clarity of the indentations in the corners. Therefore, this demineralization regimen was employed in this study.

After the pilot examination, each sample’s surface was divided into three equal areas (5 × 3 mm). The incisal third of the specimens were covered with acid-resistant nail varnish (ArtDeco, Germany) and remained intact throughout the study. The middle and bottom thirds were demineralized by immersion in the demineralization solution with 0.1 ppm NaF at 37 °C for six hours. The microhardness of four randomly selected samples confirmed a reduction of up to 100 units in microhardness down to a depth of 100 μm. The demineralized enamel was then preserved with nail varnish. The bottom third of each sample was further remineralized with varnishes following demineralization.

### Application of test agents

The samples were divided into five groups and treated accordingly (*n* = 11 each). The study groups received experimental 0.5%, 2%, and 8% w/w GA varnishes, and 2.26% fluoride varnish (V-varnish, Vericom, Seoul, Korea), while the control group received a varnish base without active ingredients.

### pH cycling

The samples underwent a pH cycling of de/remineralization regime, as described by Godoi et al. [24]. First, the specimens’ surfaces were dried, and a double layer of varnish was applied to the tooth surface. Then, all samples were placed in a remineralizing solution with the following composition for 4 h: 1 mmol/L CaCl_2_, 3 mmol/L Na_2_HO_4_, 100 mmol/L NaCl, 0.1 ppm NaF, and 0.2% sodium benzoate (pH = 6.5) [25]. Subsequently, the samples were immersed in the demineralizing solution for 2 h. In all the test groups, after removing the varnishes with distilled water and wet sterile gas, the samples were placed in the remineralization solution at room temperature for 18 h. A new layer of varnish was applied at the beginning of each cycle. Fresh solutions were prepared every two days. pH cycling continued for six days. Samples were kept in pairs throughout the pH cycling in 30 separate containers.

### Measuring microhardness

To evaluate microhardness values at depths of 30, 75, and 120 μm from the surface, a longitudinal section was made in the middle of each sample. A section with a minimum thickness of 2 mm was obtained from each sample and polished with silicon carbide papers. Each section included three areas: intact enamel, demineralized enamel, andremineralized enamel.

Microhardness measurement was conducted using a Vickers microhardness tester (MH3, Koopa Pashoohesh, Iran) under 100 g loads applied for 10 s at three different points, each 1 mm apart. The percentage surface microhardness recovery (SMHR%) at each depth was calculated as follows [21]:

$$\begin{array}{l}SMHR = (\frac{{{\rm{microhardness\ after\ remineralization}} - {\rm{microhardness\ after\ demineralization}}}}{{{\rm{baseline\ surface\ microhardness}} - {\rm{microhardness\ after\ demineralization}}}})\\\times 100\end{array}$$

### Statistical analysis

Data was analyzed using SPSS 16.0 (IBM, United States). Normal data distribution was evaluated using the Shapiro-Wilk test. An ANOVA and Tukey HSD post-hoc tests were used to compare the SMHR% between the groups. A significance level of 0.05 was considered.

## Results

Table 1 presents the average microhardness and SMHR% at various depths in intact, demineralized, and remineralized enamel. At a 30 μm depth, the group treated with 0.5% GA varnish had the highest mean SMHR% (73.98 ± 41.85%), whereas the control group displayed the lowest SMHR% (− 1.83 ± 6.50%). The 8% GA varnish group ranked second regarding the highest SMHR%. ANOVA statistical analysis revealed a significant difference between the groups in mean SMHR% at a depth of 30 μm (*p* < 0.001).

**Table 1:** Mean ± Standard Deviation (SD) of microhardness values of intact, demineralized, and remineralized enamel and microhardness recovery (%) of the study groups at different enamel depths

Depth	Varnishes	Intact enamel (Mean ± SD)	Demineralized enamel (Mean ± SD)	Remineneralized enamel (Mean ± SD)	Microhardness recovery (%)	*P* value
30 μm	0.5% gallic acid	215.38 ± 43.57	121.79 ± 34.02	185.8 ± 48.55	73.98 ± 41.85	< 0.001*
	2% gallic acid	276.25 ± 38.88	130.83 ± 37.81	164.67 ± 36.11	29.55 ± 12.21	
	8% gallic acid	278.95 ± 40.75	139.93 ± 38.82	186.74 ± 49.27	40.35 ± 25.68	
	Fluoride	263.22 ± 32.61	116.80 ± 34.01	165.46 ± 50.94	38.15 ± 10.68	
	Control	254.48 ± 28.48	115.85 ± 22.41	114.30 ± 22.88	-1.83 ± 6.60	
75 μm	0.5% gallic acid	244.46 ± 36.36	189.93 ± 45.43	222.60 ± 42.53	40.18 ± 90.28	0.264
	2% gallic acid	277.88 ± 27.53	216.17 ± 58.05	217.15 ± 46.71	41.09 ± 15.75	
	8% gallic acid	296.98 ± 31.08	227.52 ± 58.18	251.55 ± 39.52	23.02 ± 28.40	
	Fluoride	275.61 ± 30.91	205.83 ± 36.52	221.49 ± 40.27	38.87 ± 35.13	
	Control	271.32 ± 29.56	213.06 ± 30.65	203.01 ± 55.29	30.63 ± 11.40	
120 μm	0.5% gallic acid	270.51 ± 41.51	231.65 ± 46.37	261.04 ± 31.35	74.89 ± 5.66	0.065
	2% gallic acid	290.09 ± 21.76	257.20 ± 50.50	256.83 ± 27.03	67.82 ± 58.01	
	8% gallic acid	307.33 ± 35.20	271.85 ± 62.19	281.65 ± 51.54	22.10 ± 61.69	
	Fluoride	287.42 ± 27.22	265.22 ± 24.39	268.96 ± 22.83	21.66 ± 41.81	
	Control	287.41 ± 22.76	261.88 ± 31.17	230.11 ± 34.73	20.71 ± 70.52	

*A significant difference was observed between the microhardness values of different groups based on the ANOVA test (*p* < 0.05)

Subsequent post-hoc analysis using the Tukey HSD test showed that the control group’s SMHR% was significantly lower than the intervention groups’ SMHR% at a depth of 30 μm (*p* < 0.05). Moreover, the 0.5% GA varnish group had a significantly higher SMHR% than the 2% GA varnish group (*p* < 0.001), the 8% GA varnish group (*p* = 0.008), and the fluoride varnish group (*p* = 0.006). There was no significant difference in the mean SMHR% among the 2% GA varnish, 8% GA varnish, and fluoride varnish groups.

At a 75 μm depth, the 0.5% GA varnish group displayed the highest mean SMHR% (64.18 ± 90.28%), while the control group showed the lowest SMHR% (63.40 ± 30.11%). However, the ANOVA test revealed no statistically significant difference in mean SMHR% among the groups at this depth.

Likewise, at a 120 μm depth, the 0.5% GA varnish group exhibited the highest SMHR% (74.89 ± 15.66%), while the control group had the lowest SMHR% (20.71 ± 70.52%). Nonetheless, as with the 75 μm depth, there was no statistically significant difference in the mean SMHR% among the groups.

## Discussion

The findings of this study indicate that enamel microhardness improved in all intervention groups at a depth of 30 μm. Notably, the 0.5% GA varnish showed the highest microhardness recovery at all depths, especially at a depth of 30 μm. 2% and 8% GA varnishes showed comparable microhardness recovery ability to fluoride varnishes.

Several studies have investigated the remineralizing mechanism of GA. It has been reported that GA promotes hydroxyapatite crystal growth along the c-axis [17]. GA pyrogallol group and quinone form a strong bond with the NH2 and SH groups of enamel proteins and Ca, which leads to hydroxyapatite crystal nucleation and reduced demineralization [17, 26–28]. To our knowledge, no previous studies have used GA varnishes, and most studies have examined the remineralizing effects of plant extracts containing GA, such as grape seeds and *Galla chinensis*, or its solution.

Hameed et al. [29] reported no significant differences in enamel surface microhardness and calcium/phosphate ratio between using a 0.56% grape seed extract solution and a 1000 ppm sodium fluoride solution after pH cycling. However, Chu et al. [30] and Cheng et al. [8] found that a 0.4% GA solution and 0.4% *Galla chinensis* had less remineralizing effects than a 1000 ppm sodium fluoride solution. In a rat model, Zhang et al. [31] discovered that enamel mineral density was higher after using sodium fluoride compared to Galla chinensis and GA. Given the positive impact of GA on dental hard tissue mineralization and the inconsistency in studies regarding its efficacy relative to fluoride, this study evaluated the effectiveness of experimental GA varnishes compared to fluoride varnish. However, due to variations in GA forms (varnish vs. extract) and the presence of other polyphenol compounds in plant extracts, a direct comparison of the current study’s findings with most other studies was not feasible.

Surface microhardness testing was chosen for this study because of its ease, reliability, non-destructiveness, and speed [32]. Featherstone et al. [33] found a correlation between microhardness values and mineral ratios in carious lesions, suggesting that this test is sensitive enough to detect early enamel demineralization. A unique feature of the current study is the exploration of microhardness at three distinct depths, highlighting the impact of varnishes across varying enamel depths, in contrast to most other studies that primarily focus on surface microhardness.

Most studies on remineralizing varnishes have been conducted on primary teeth. In the current study, pilot experiments were conducted to determine the optimal timing and composition of the demineralization solution for bovine teeth, which can be useful for future researchers due to the challenges in collecting intact deciduous teeth and their limited surface area.

In this study, the maximum SMHR% in the intervention groups was observed at a depth of 30 μm, while SMHR% decreased at higher depths. This finding may be attributed to the fact that mineral deposition primarily occurs on the surface, reducing calcium and phosphorus ions’ penetration into the inner layers [9]. According to Cheng et al. [9], treatment with a 0.4% GA solution resulted in hydroxyapatite crystals with dimensions averaging 24.50 nm, similar to sodium fluoride (25.05 nm) but larger than intact enamel crystals (22.94 nm). However, the surface porosity following GA solution application was lower than 1000 ppm fluoride, deionized water, and 0.4% aqueous extract of *Galla chinensis*, and resembled the surface porosity after treatment with sodium fluoride. Cheng et al. [8] also found that surface minerals in the 0.4% GA solution group were higher than in the body of the primary carious lesion.

The average SMHR% of the 0.5% GA varnish at a depth of 30 μm was approximately 74%. The varnish formulation in this study allowed GA to adhere to the enamel surface, potentially facilitating remineralization by providing a high calcium concentration adjacent to the tooth surface. Previous studies by Gao et al. [34] and Chu et al. [21] reported enamel surface SMHR% of 17.5% and 14.8%, respectively, after 12 days of pH cycling and treatment with a 0.4% aqueous GA solution. The higher SMHR% in this study could be attributed to differences in GA form (varnish vs. aqueous solution), higher GA concentration, larger sample size, and differences in the pH cycling design. Chu et al. [21] performed pH cycling for a more extended period (12 days) with four one-minute immersions in the tested solutions, which may have led to a lower SMHR% due to GA dissolution. Additionally, the remineralization solution in this study, unlike Chu et al. [21] study, contained 0.1 ppm sodium fluoride, which has shown synergistic effects with GA [10].

The concentration of GA can influence the intensity of hydroxyapatite crystallization. The 0.5% GA varnish group showed greater SMHR% compared to the 2% and 8% GA varnish groups. The reason might be attributed to the decreased crystallization rate of hydroxyapatite crystals with higher GA concentrations. According to Jerdioui et al. [18] and Tang et al. [35], increasing GA concentration inhibits nanohydroxyapatite crystals’ precipitation and nucleation. In turn, high GA concentration results in highly amorphous calcium particles, which could explain lower enamel SMHR% when higher concentrations of GA were applied to the surface. Tang et al. [36] found that in the presence of 4 g/L GA, the hydroxyapatite crystals’ growth rate decreased, and their dimensions also became smaller. These crystals formed rod-shaped structures on the seventh day and turned into sea urchin-like structures on the fourteenth day. The pH cycling in this study was continued for six days, and it is possible that over a more extended period, the SMHR% might increase in the groups with higher GA levels.

The SMHR% of the fluoride varnish in this study was 38.15, which is approximately similar to the results of the Siqueira et al. [37] study, which found the SMHR% after using Duraphat 2.26% fluoride varnish (Colgate) to be 36%. However, they analyzed the mean microhardness values ranging from 10 to 220 μm on the outer enamel surface and conducted pH cycling for 8 days. This could potentially account for the lower SMHR% observed in their study. In the current study, the SMHR% of the 0.5% GA varnish at a depth of 30 μm was approximately twice that of the 2.26% fluoride varnish. This difference may be attributed to the different remineralization mechanisms of GA and fluoride. GA likely acts as a calcium ion carrier and facilitates remineralization by bonding with proteins and hydroxyapatite crystals [16].

One significant limitation of this study is its in vitro nature, which may impact the clinical effectiveness of the experimental remineralization varnishes. Further clinical studies evaluating the oral health indicators and solubility of the 0.5% GA varnish in saliva are recommended. Additionally, future research should explore the varnish’s antibacterial properties against cariogenic bacteria and pathogens associated with periodontal diseases. Previous studies have indicated that remineralization may offer protection against erosive enamel wear [38]. Thus, research into the potential protective effects of 0.5% GA varnish on enamel wear is warranted. Moreover, the type of potentially formed crystals on the enamel surface layers was not assessed, which requires further investigation.

In conclusion, the current in vitro study showed that the remineralization ability of 0.5% GA varnish was higher than 2.26% fluoride varnish, especially at superficial layers. GA varnishes with higher GA concentrations showed remineralization efficacy comparable to the fluoride varnish. Therefore, given GA’s favorable properties and non-toxic nature, the experimental GA varnish shows promise as a viable substitute for fluoride varnish in caries prevention, pending further clinical investigations.

## Data Availability

The datasets used and/or analyzed during the current study are available from the corresponding author on reasonable request.
